# Bioelectrochemical methanation by utilization of steel mill off-gas in a two-chamber microbial electrolysis cell

**DOI:** 10.3389/fbioe.2022.972653

**Published:** 2022-09-09

**Authors:** Sabine Spiess, Amaia Sasiain Conde, Jiri Kucera, David Novak, Sophie Thallner, Nina Kieberger, Georg M. Guebitz, Marianne Haberbauer

**Affiliations:** ^1^ K1-MET GmbH, Linz, Austria; ^2^ Department of Biochemistry, Faculty of Science, Masaryk University, Brno, Czechia; ^3^ Voestalpine Stahl GmbH, Linz, Austria; ^4^ ACIB GmbH (Austrian Centre of Industrial Biotechnology), Graz, Austria; ^5^ Department of Agrobiotechnology, Institute of Environmental Biotechnology, University of Natural Resources and Life Sciences Vienna, Tulln an der Donau, Austria

**Keywords:** bioelectrodes, metagenomic analysis, electromethanogenesis, microbial electrolysis cell, exhaust gas

## Abstract

Carbon capture and utilization has been proposed as one strategy to combat global warming. Microbial electrolysis cells (MECs) combine the biological conversion of carbon dioxide (CO_2_) with the formation of valuable products such as methane. This study was motivated by the surprising gap in current knowledge about the utilization of real exhaust gas as a CO_2_ source for methane production in a fully biocatalyzed MEC. Therefore, two steel mill off-gases differing in composition were tested in a two-chamber MEC, consisting of an organic substrate-oxidizing bioanode and a methane-producing biocathode, by applying a constant anode potential. The methane production rate in the MEC decreased immediately when steel mill off-gas was tested, which likely inhibited anaerobic methanogens in the presence of oxygen. However, methanogenesis was still ongoing even though at lower methane production rates than with pure CO_2_. Subsequently, pure CO_2_ was studied for methanation, and the cathodic biofilm successfully recovered from inhibition reaching a methane production rate of 10.8 L m^−2^d^−1^. Metagenomic analysis revealed *Geobacter* as the dominant genus forming the anodic organic substrate-oxidizing biofilms, whereas *Methanobacterium* was most abundant at the cathodic methane-producing biofilms.

## Introduction

Global warming caused by anthropogenic greenhouse gas emissions is becoming a serious problem for the environment and all forms of life (Bajracharya et al., 2017). Carbon dioxide (CO_2_), as one of the main contributors of greenhouse gases, is mainly released by human activities such, as burning fossil fuels or deforestation (Pachauri and Meyer, 2014). These activities have increased the atmospheric CO_2_ concentrations annually since the industrial age, and half of the CO_2_ emissions between 1750 and 2011 have occurred in the last 40 years (Pachauri and Meyer, 2014). To combat global warming CO_2_ mitigation technologies, such as carbon capture and storage (CCS) and carbon capture and utilization (CCU), have been proposed as strategies (Bian et al., 2020). Therefore, biological CO_2_ conversion with microbial electrolysis cells (MECs) attracted great interest. Bioelectrochemical systems are based on the electroactivity of microorganisms attached to the electrode(s), which release electrons to the anode during substrate oxidation or receive electrons from the cathode during substrate reduction (Rabaey and Rozendal, 2010). MEC technology combines CO_2_ reduction with the production of versatile chemical compounds, such as methane (CH_4_), acetate, and ethanol, supported by a low energy input (Zhang and Angelidaki, 2014; Kadier et al., 2016). In particular, the production of CH_4_ attracts attention, because it is thermodynamically the most favorable product for CO_2_ reduction (Eq. 1) (Jiang et al., 2019).

$$CO_{2}+8H^{+}+8e^{-}\to CH_{4}+2H_{2}O\;\;\;–0\mathrm{.244 V}\;\mathrm{vs}\mathrm{. SHE}$$ (1)

**FIGURE 1:**
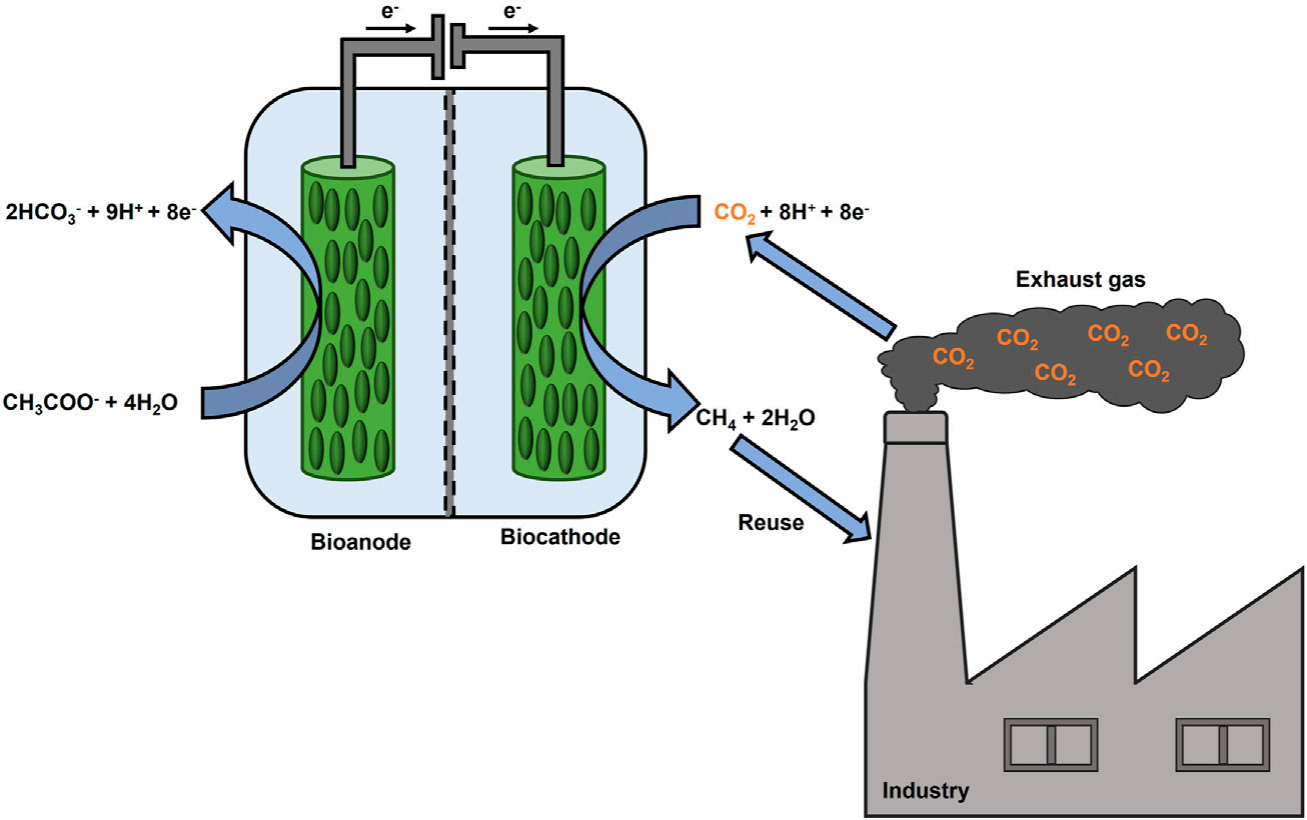
Scheme of a fully biocatalyzed electrochemical system to produce CH_4_ from CO_2_ rich exhaust gas.

Methanogenic archaea can use CO_2_, as a terminal electron acceptor, and hydrogen (H_2_), as an electron source, to produce CH_4_ as a metabolic product (Bajracharya et al., 2017). These microbes serve as biocatalysts for CO_2_ reduction in MECs, a process referred to as electromethanogenesis (Cheng et al., 2009). Electromethanogenesis can proceed *via* direct extracellular electron transfer or indirectly *via* (bio)electrochemically produced H_2_, formate or acetate (Van Eerten-Jansen et al., 2015). An oxidation reaction at the anode supplies electrons and protons for a reduction reaction. For instance, water oxidation can occur at the anode of a MEC. However, also an organic substrate-oxidizing bioanode can be coupled to a CH_4_-producing biocathode to reduce the external power supply (Villano et al., 2010) as shown in Figure 1. Recently, an increasing number of research groups have been focusing on this energy-efficient fully biocatalyzed MEC, investigating the effects of the anodic potential settings (Villano et al., 2016), testing the sequential polarization of the anodic and cathodic chambers (Zeppilli et al., 2019), as well as electrode modifications and their effect on performance parameters (Seelajaroen et al., 2020). Lately, microbial electrosynthesis from unpurified CO_2_ from brewery industry was tested for the first time within a bioelectrochemical reactor, consisting of a biotic cathode and an abiotic anode, producing acetate at a rate of 0.26 g L^−1^ d^−1^ (Roy et al., 2021). Also, real exhaust gas from a coal-fired power plant was used as a feedstock for lycopene production in a single bioelectrochemical reactor (Wu et al., 2022). On the other hand, to the best of our knowledge, exhaust gas has not been studied in a fully biocatalyzed CH_4_ producing MEC so far. Therefore, the present study aimed to explore the CH_4_ production by utilization of CO_2_ rich exhaust gas in a MEC, consisting of a bioanode and a biocathode, and to investigate how exhaust gas composition may influences the microbial activity. Two H-type MECs, referred to as MEC1 and MEC2, were setup. MEC1 was flushed with pure CO_2_, whereas in MEC2 the usage of exhaust gas was examined. Furthermore, prokaryotes on bioelectrodes of MEC2 were identified after exhaust gas flushing and were compared to MEC1, which was flushed with pure CO_2_, to discuss microbial community changes due to exhaust gas flushing. As steel production is one of the most energy-intensive processes, emitting 7% of the global CO_2_ emissions (Holappa, 2020), steel mill off-gas was selected as the exhaust gas. Two steel mill off-gases differing in composition were tested in MEC2 by applying a constant potential on the bioanode. Setting the anode potential at a fixed value offers some advantages as described recently (Villano et al., 2016). First, it leads to a faster start-up regarding organic substrate degradation as well as current generation. Second, fixing the anode potential regulates the biological activity and underlying electron transfer mechanisms of microorganisms (located on both electrodes). Furthermore, the cathode potential will get adjusted to sustain the current generating anode. Throughout all experiments process parameters, such as chemical oxygen demand (COD) removal and CH_4_ production, were monitored. Further, Coulombic efficiencies and energetic parameters were calculated. Also, the CH_4_ production rate was compared with other studies, which investigated two-chamber CH_4_ producing MECs.

## Materials and methods

### MEC setup

The experiments were performed in two-chamber H-cells with a working volume of 220 ml each, separated by a pretreated proton exchange membrane (Nafion 117, Chemours, Wilmington, DE, United States), as previously described (Spiess et al., 2021). Two MECs were set up and referred to as MEC1 and MEC2. Carbon felt (projected surface area 15 cm^2^, Alfa Aesar, Heysham, United Kingdom) was selected as an electrode material. Prior to use, the carbon felt electrodes were pretreated with isopropanol and hydrogen peroxide as described elsewhere (Spiess et al., 2021). Titanium wires (0.25 mm, Alfa Aesar, Heysham, United Kingdom) were used to enable the external electrical connection. Ag/AgCl reference electrodes were placed in both MEC chambers. All voltages reported in this study are with respect to Ag/AgCl reference electrode (3 M NaCl, +209 mV vs. standard hydrogen electrode). 200 ml phosphate buffer solution (PBS, pH 7.2) was used as an electrolyte for both chambers, consisting of the following components (per liter): 3 g KH_2_PO_4_, 2.5 g K_2_HPO_4_, 0.13 g NaCl, 0.31 g NH_4_Cl, 6 g NaHCO_3_, 0.04 g MgSO_4_.7H_2_O, 12.5 ml trace element solution SL 10 (DSMZ 320), and 5 ml vitamin solution (DSMZ 141). All MEC chambers were inoculated with 20 ml anaerobic digester sludge collected from a wastewater treatment plant. Prior to inoculation, solid contaminants were removed by centrifugation at 2,150 *g* for 10 min. All experiments were performed at room temperature, and the anolytes and catholytes were continuously mixed at 70 rpm using a magnetic stirrer IKA RCT basic (Staufen, Germany).

### MEC operation

During the experiments, the anode chambers of MEC1 and MEC2 were supplied with acetate (1 g L^−1^) three times per week, whereas the cathode chambers were flushed with pure CO_2_ (99.995 vol%) or steel mill off gas (SMO). 100% of the anolyte and 90% of the catholyte were replaced with fresh PBS at each feeding. Before each feeding, liquid and gas samples were taken from anode and cathode chambers, respectively. MEC bioanodes were maintained under anaerobic conditions during all experiments by flushing with pure CO_2_ after each feeding. Experimental procedures are summarized in Table 1. During adaptation MECs were operated in duplicate by applying a constant potential of +400 mV vs. Ag/AgCl on the anode using a PM-100 potentiostat (Jaissle Elektronik GmbH, Münster, Germany), and the CH_4_ production using pure CO_2_ for flushing the biocathode was evaluated. In experiment 1 an exhaust gas was tested in MEC2 by purging the cathode at each feeding with SMO, whereas MEC1 was flushed with pure CO_2_ as a control. A total of six feeding cycles were repeated. The composition of the first steel mill off-gas (SMO-1) used was as follows: 73.4 vol% N_2_, 22 vol% CO_2_, and 4.6 vol% O_2_. Afterwards, the catholyte from MEC2 was flushed again with pure CO_2_ instead of exhaust gas for six feeding cycles. During adaptation and experiment 1 an anodic potential of +400 mV vs. Ag/AgCl was set, as a high potential probably leads to a faster start-up time and to a thicker biofilm formation as described elsewhere (Wagner et al., 2010). Finally, the biofilms were scraped from all MEC1 and MEC2 bioelectrodes, resuspended in Tris-EDTA buffer (pH 8.0), and frozen at −80°C. In experiment 2 the applied anode potential was reduced from +400 mV to +300 mV vs. Ag/AgCl in MEC1 and MEC2, and was tested for eight feeding cycles to investigate the effects of a reduced potential on CH_4_ production rates. The CH_4_ production by utilization of the second steel mill off-gas (SMO-2) was investigated in experiment 3 in MEC2 by applying a constant anode potential of +300 mV vs. Ag/AgCl for six feeding cycles. The composition of SMO-2 used was as follows: 74.2 vol% N_2_, 23.1 vol% CO_2_, and 2.7 vol% O_2_. MEC1 served as a control during this experiment and was purged with pure CO_2_. During the experiments the cathode potentials of MECs were monitored once per day with a Voltcraft VC880 multimeter (Hirschau, Germany).

**TABLE 1:** Overview of the experimental conditions of MEC1 and MEC2.

Experimental conditions	Reactors	Applied potential vs. Ag/AgCl	Cycles	Anode chamber feeding	Cathode chamber flushing
Adaptation	MEC1 and MEC2	+400 mV	Approx. 3 months	1 g L^−1^ acetate	CO_2_
1	MEC1 (control)	+400 mV	12	1 g L^−1^ acetate	CO_2_
	MEC2	+400 mV	6	1 g L^−1^ acetate	SMO-1
	MEC2	+400 mV	6	1 g L^−1^ acetate	CO_2_
2	MEC1 and MEC2	+300 mV	8	1 g L^−1^ acetate	CO_2_
3	MEC1 (control)	+300 mV	6	1 g L^−1^ acetate	CO_2_
	MEC2	+300 mV	6	1 g L^−1^ acetate	SMO-2

### Analytics and calculations

A COD test was used to estimate the content of organic compounds as previously described (Spiess et al., 2021). The COD removal efficiency was calculated according to Eq. 2 in which *∆COD* represents the depleted COD, and *COD*_*IN*_ is the COD of a provided substrate.

$$COD\;removal\;efficiency\;\left(\%\right)=\frac{\Delta COD}{COD_{IN}}\times 100$$ (2)

The CH_4_ production in MECs was analyzed by injecting a 2 ml sample from the cathode’s headspace into a 6890 GC system (Agilent Technologies, Santa Clara, CA, United States), equipped with a flame ionization detector and a series-connected helium ionization detector. The Coulombic efficiency (CE) was calculated according to Eq. 3, in which *V*_*CH4*_ represents the CH_4_ production in m³, eight electrons are required to reduce CO_2_ to CH_4_, *F* is the Faraday constant (96,485 C mol^−1^), *V*_*m*_ is the molar volume (0.0252 m³ mol^−1^), *I* represent the recorded current, and *t* means the time.

$$CE\;cathode\left(\%\right)=\;\frac{V_{CH4}\times 8\times F}{Vm\times \int_{0}^{t}Idt}\times 100$$ (3)

The energy consumptions for COD removal (kWh kg^−1^ COD) and CO_2_ removal (kWh Nm^−^³ CO_2_) were calculated for standard conditions as described elsewhere (Geppert et al., 2016; Zeppilli et al., 2019). The energy efficiency (ηE) was calculated according to Eq. 4, in which *ΔG*_*CH4*_ represents the Gibbs free energy of CH_4_ oxidation (890.4 kJ mol^−1^), while *V*_*CH4*_ is the CH_4_ production in m³, *V*_*m*_ (0.0252 m³ mol^−1^) is the molar volume, *E*_*Cell*_ is the cell voltage, *I* represent the recorded current, and *t* means the time.

$$\text{ηE}=\frac{-\Delta G_{CH4}\times V_{CH4}}{V_{m}\times E_{Cell}\times \int_{0}^{t}Idt}$$ (4)

### Metagenomic analysis

Microbial DNA was isolated using the DNeasy UltraClean Microbial Kit (Qiagen, Germany), according to the manufacturer’s instructions. The hypervariable V4 region of 16S rRNA was selected to analyze prokaryotes, and the gene encoding the *α*-subunit of methyl coenzyme M reductase (*mcrA*) was used for the analysis of methanogenic archaea. The hypervariable region V4 was amplified with unique barcoded oligonucleotides 515F and 806R, as previously described (Spiess et al., 2021). The *mcrA* gene was amplified with gene-specific oligonucleotides qmcrA-F and mcrA-rev (Denman et al., 2007; Steinberg and Regan, 2008) containing Illumina adapter overhang nucleotide sequences (Supplementary Table S1). PCR amplification was performed using Platinum II Taq Hot-Start DNA polymerase (Thermo Fisher Scientific, United States), as follows: initial DNA denaturation step at 95°C for 3 min, 25 cycles of DNA denaturation at 94°C for 20 s, annealing at 60°C for 30 s with a 50% thermal ramp, extension at 72°C for 30 s, and a final extension step at 72°C for 5 min. The amplification products were purified using the UltraClean PCR Clean-Up Kit (Qiagen, Germany), according to the manufacturer’s instructions. Each purified PCR sample was tagged with sequencing adapters using the Nextera XT Indexes Kit (Illumina, United States) and KAPA HiFi HotStart Readymix PCR Kit (Kapa Biosystems, United States), according to the manufacturer’s specifications. The indexed products were purified by Agencourt^®^ AMPure XP beads (Beckman Coulter, United States) according to Illumina recommendations with a final elution step of 25 μl to maintain a high concentration of amplicons. Samples were quantified using a Qubit 4.0 fluorometer (Thermo Fisher Scientific, United States), followed by manual normalization to the lowest observed concentration and pooling to create the final library. The Fragment Analyzer (Advanced Analytical Technologies, United States) was then used to determine the quality of the library. The library was sequenced using a MiniSeq System (Illumina, United States) with MiniSeq Mid Output Kit (300 cycles). Raw fastq reads were processed in R software (4.0.3) using the open-source package DADA2 (1.16.0), as previously described (Spiess et al., 2021). 16S rRNA gene sequences were analyzed using the Silva database (Quast et al., 2013), while a *mcrA* ARB database was used to analyze *mcrA* sequences (Angel et al., 2012). The summaries of all 16S and *mcrA* amplicon sequence variants (ASVs) are shown in Supplementary Tables S2, S3, respectively. Datasets generated and analyzed during this study are available in the NCBI Sequence Read Archive under project number BioProject ID: PRJNA782972.

## Results and discussion

### Methane production by utilization of SMO-1

**FIGURE 2:**
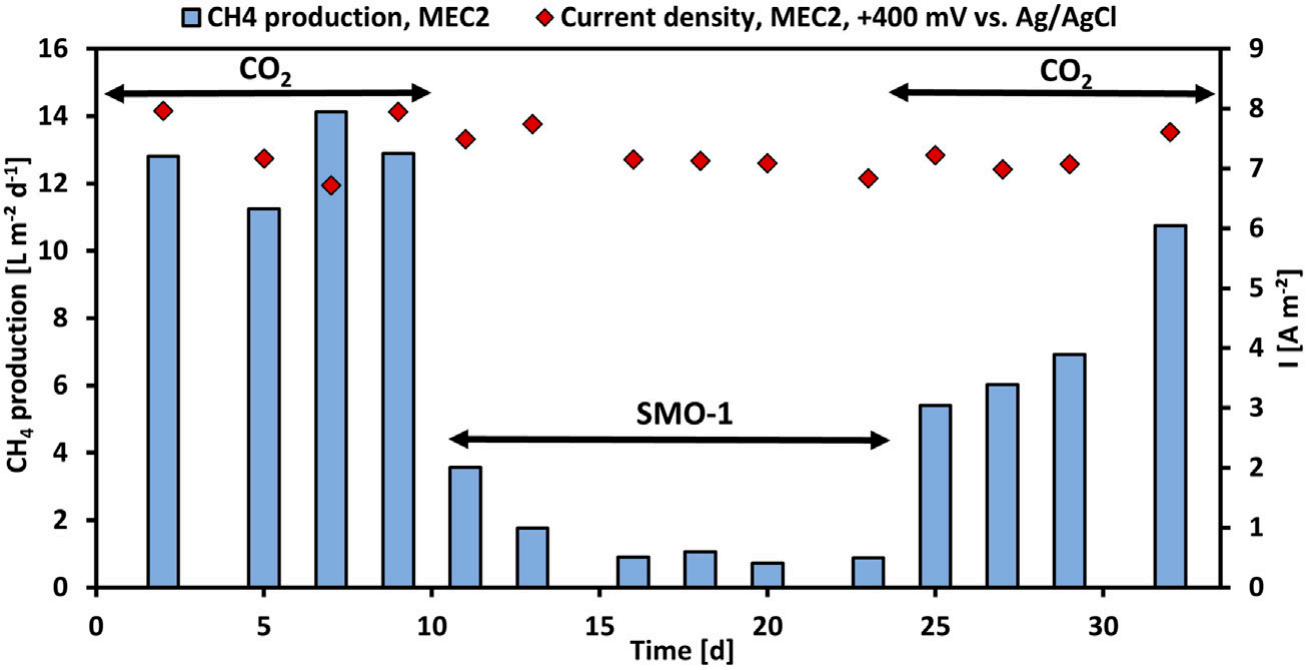
CH_4_ production per projected electrode surface area and day (blue bars) and current density per projected electrode surface area (red rhombuses) of MEC2 before and after flushing with SMO-1 vs. operation time.

First, the CH_4_ production using pure CO_2_ was investigated in MEC2, then the CH_4_ production with exhaust gas was evaluated. Thus, the cathode chamber of MEC2 was flushed with SMO-1 at each feeding, MEC1 served as a control during this experiment. Figure 2 illustrates the CH_4_ production per projected electrode surface area and day and the current density per projected electrode surface area of MEC2. Flushing MEC2 with pure CO_2_ resulted in a maximum CH_4_ production rate of 14.1 L m^−2^ d^−1^. However, after the first flushing of the MEC2 biocathode with SMO-1 the CH_4_ production dropped immediately to 3.6 L m^−2^ d^−1^. After the second flushing the CH_4_ production was further halved to 1.8 L m^−2^ d^−1^. The CH_4_ production significantly decreased after flushing with SMO-1 (*p* < 0.001; *t-*test) and subsequently stabilized at an average of 0.9 L m^−2^ d^−1^. The current density during this experiment was in a range of 6.7–8.0 A m^−2^. The cathode potential of MEC2 was on average -1,180 ± 54 mV during SMO-1 and -1,151 ± 66 mV during CO_2_ flushing. The significant reduction in CH_4_ production was most likely due to the elevated O_2_ concentration (4.6 vol%) in SMO-1, which most likely caused strong inhibition of anaerobic methanogens. In addition, GC analysis of the cathodic headspace samples revealed a threefold increase in the H_2_ concentration, but no further conversion to CH_4_. However, it is noticeable that methanogenesis was still ongoing in the presence of O_2_ from SMO-1 even though at lower CH_4_ production rates than before.

After six cycles using SMO-1 the MEC2 biocathode was flushed again with pure CO_2_. A significant increase in CH_4_ production was observed again during CO_2_ re-flushing (*p* < 0.001; *t-*test). The CH_4_ production increased immediately to 5.4 L m^−2^ d^−1^ and continued to rise steadily up to 10.8 L m^−2^ d^−1^ at day 32 (Figure 2). In addition, CH_4_ production differed significantly during the first and second purging with pure CO_2_ (*p* < 0.01; *t-*test). However, the elevated recovery of CH_4_ production rate of the biocathode after the re-flushing with pure CO_2_ suggested a return to the initial values after day 32. Thus, the CH_4_-producing biofilm adhering to the MEC2 biocathode successfully recovered from O_2_ inhibition caused by flushing with SMO-1. Although methanogens are strict anaerobic, mixed methanogenic cultures may be able to tolerate a certain amount of O_2_ for a short time (Zitomer and Shrout, 2000). As MEC2 was inoculated with a mixed culture from sewage sludge and has been adapted for approximately 3 months before exhaust gas was flushed, a very robust biofilm may have developed on the cathode. Nevertheless, the biofilm got inhibited during SMO-1 flushing. However, probably due to the long adaptation and the mixed inoculum this concentration of O_2_ did not harm methanogens irreversible.

Table 2 compares the CH_4_ production of MEC2 from this study (when flushed with pure CO_2_ at +400 mV vs. Ag/AgCl) with other studies. The CH_4_ production rates were found to range widely from 0.017 to 0.678 mmoL L^−1^ h^−1^. On the other hand, the cathodic CE showed higher similarity among all studies, ranging from 55 to 74%. The MEC operating conditions differ in potentiostatic control of the anode or cathode. If the cathodic potential was controlled, the CH_4_ production rates ranged from 0.035 to 0.678 mmoL L^−1^ h^−1^, whereas controlling the anode showed lower production rates varying from 0.017 to 0.339 mmoL L^−1^ h^−1^. Thus, the result of this study (0.150 mmoL L^−1^ h^−1^) was within the range of other studies. If an organic substrate oxidizing-bioanode is coupled to a CH_4_-producing biocathode, electromethanogenesis seems to strongly depend on the amount of organic substrate provided, accompanied by COD removal efficiency and the produced current density. In our previous study, the COD of the supplied substrate was 600 mg L^−1^ (fed twice weekly), resulting in a CH_4_ production of 0.018 mmol L^−1^ h^−1^ (Spiess et al., 2021). Whereas, in this study, the bioanode was provided with 1,000 mg COD L^−1^ (three times weekly), resulting in an eightfold higher CH_4_ production rate (0.150 mmol L^−1^ h^−1^).

**TABLE 2:** Comparison of two-chamber CH_4_ producing MECs.

Electrode material	Working electrode	Working electrode potential	V_CH4_ [mmol L^−1^ h^−1^]	CE cathode [%]	References
Carbon felt	Cathode	-0.85 V vs. Ag/AgCl	0.075	60.9	Jiang et al. (2013)
Carbon felt	Cathode	-0.9 vs. Ag/AgCl	0.157 ± 0.014	60.90 ± 2.27	Yang et al. (2018)
Graphite rod	Cathode	-0.9 V vs. SHE	0.678	74 ± 5	Zeppilli et al. (2019)
NR-modified carbon felt	Cathode	−1.0 V vs. Ag/AgCl	0.058 ± 0.007	58.90 ± 11.47	Yang et al. (2020)
AQDS-modified carbon felt	Cathode	−1.0 V vs. Ag/AgCl	0.035 ± 0.010	60.88 ± 4.01	Yang et al. (2020)
Graphite felt	Cathode	−1.0 V vs. Ag/AgCl	0.094	55	Mateos et al. (2020)
Graphite granules	Anode	+0.5 V vs. SHE	0.031	57	Villano et al. (2011)
Graphite rod	Anode	+0.2 V vs. SHE	0.339	61 ± 5	Zeppilli et al. (2019)
Chitosan-modified carbon felt	Anode	+0.4 V vs. Ag/AgCl	0.017	57	Seelajaroen et al. (2019)
Isopropanol pretreated carbon felt	Anode	+0.4 V vs. Ag/AgCl	0.018	58	Spiess et al. (2021)
Isopropanol pretreated carbon felt	Anode	+0.4 V vs. Ag/AgCl	0.150	63	This study

### Effect of the applied anode potential

**FIGURE 3:**
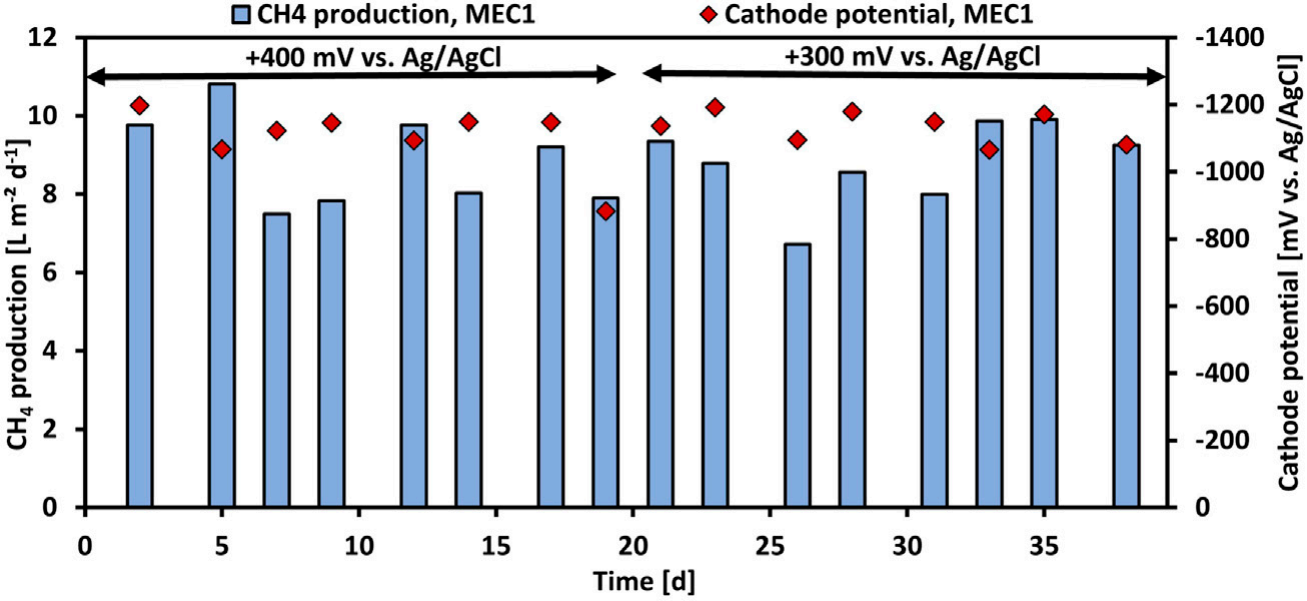
CH_4_ production per projected electrode surface area and day (blue bars) and monitored cathode potentials (red rhombuses) of MEC1 at an applied anode potential of +400 mV and +300 mV vs. Ag/AgCl during CO_2_ flushing vs. operation time.

In experiment 2 the effect of the applied anode potential on the CH_4_ production when pure CO_2_ was flushed was investigated. Therefore, the anode potential of MEC1 was reduced from +400 mV to +300 mV vs. Ag/AgCl. Figure 3 illustrates the CH_4_ production and the cathode potential of MEC1 at +400 mV and +300 mV vs. Ag/AgCl, respectively. The CH_4_ production at +400 mV ranged between 7.5 L m^−2^ d^−1^ and 10.8 L m^−2^ d^−1^, and at +300 mV between 6.7 L m^−2^ d^−1^ and 9.9 L m^−2^ d^−1^, respectively. The difference in CH_4_ production by applying an anode potential of either +400 or +300 mV was insignificant (*p* > 0.05; *t-*test). The cathode potentials of MEC1 fluctuated at an applied potential of +400 mV from −883 mV to -1,198 mV, and at +300 mV from -1,066 mV to -1,192 mV vs. Ag/AgCl. The average cathodic potential was -1,101 ± 90 mV and -1,134 ± 45 mV at +400 mV and +300 mV vs. Ag/AgCl, respectively. Also, previous mentioned results from MEC2 (1,151 ± 66 mV during CO_2_ flushing) are in a similar range and suggest a stable cathode potential during the experiments.

The performance parameters of MEC1 at applied anode potentials of +400 and +300 mV vs. Ag/AgCl are summarized in Table 3. The average CH_4_ production remained nearly constant at 8.8 L m^−2^ d^−1^ despite the reduced potential. In contrast at an applied potential of +300 mV, a higher current density, and COD removal efficiency of 6.4 A m^−2^ and 53%, respectively, were observed. The cathodic CE dropped from 65% at +400 mV to 61% at +300 mV. As the CH_4_ production remained constant despite the reduced applied potential and the current density and COD removal efficiency increased, an applied anode potential of +300 mV vs. Ag/AgCl was used for all further experiments for both MECs. Furthermore, the CH_4_ production in the bioanode of MEC1 was nominal (<1% CH_4_ was detected in the anodic headspace). This may be related to the slightly acidic pH (approx. 6.2) of the anolyte, which was measured after each feeding cycle, because methanogens are known to be inhibited easily at pH values < 6.3 and >7.8 (Chae et al., 2010).

**TABLE 3:** Comparison of monitored parameters of MEC1—COD removal efficiency, current density, CH_4_ production and CE cathode.

Parameters MEC1	+400 mV vs. Ag/AgCl			+300 mV vs. Ag/AgCl		
COD removal efficiency [%]	47	±	14	53	±	15
Current density [A m^−2^]	5.6	±	0.7	6.4	±	0.7
CH_4_ production [L m^−2^ d^−1^]	8.9	±	1.1	8.8	±	1.0
CE cathode [%]	65	±	7	61	±	7

### Methane production by utilization of SMO-2

**FIGURE 4:**
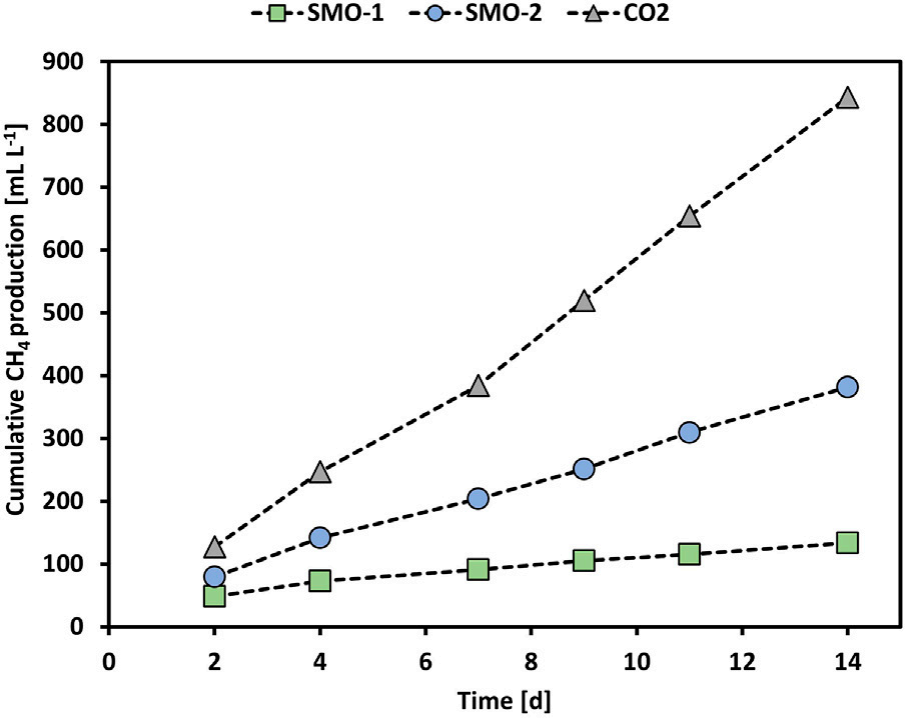
Cumulative CH_4_ production of MEC2 when flushed with pure CO_2_ (grey triangles), SMO-1 (green squares), and SMO-2 (blue circles).

Furthermore, the methanation of SMO-2 was tested in MEC2 by applying an anode potential of +300 mV vs. Ag/AgCl. However, the O_2_ concentration in SMO-2 was only 2.7 vol% compared to 4.6 vol% in SMO-1. Figure 4 shows the cumulative CH_4_ production of the MEC2 biocathode when flushed with pure CO_2_, or SMO-1 and SMO-2 containing 4.6 vol% and 2.7 vol% O_2_, respectively. The cumulative CH_4_ production during pure CO_2_ flushing was significantly higher than during SMO-1 and SMO-2 flushing (*p* < 0.001; ANCOVA). In addition, a significant difference in CH_4_ production was observed between SMO-1 and SMO-2 (*p* < 0.001; ANCOVA). The highest cumulative CH_4_ production (843 ml L^−1^) was achieved when the biocathode was flushed with pure CO_2_ and the lowest (133 ml L^−1^) when purged with SMO-1 containing 4.6 vol% O_2_ (here an anode potential of +400 mV vs. Ag/AgCl was applied). During the experiment with SMO-2, containing 2.7 vol% O_2_, the cumulative CH_4_ production was nearly three times higher (382 ml L^−1^) than with SMO-1. The obtained results demonstrated that the O_2_ concentration in the exhaust gas strongly affected CH_4_ production in MEC2 due to the inhibition of anaerobic methanogens. As well-known methanogens are strictly anaerobic and very sensitive to even low levels of O_2_ (Garcia et al., 2000), hence higher CH_4_ production rates can be expected at lower O_2_ concentrations in exhaust gas streams. Moreover, it is also possible that during SMO flushing other products/intermediates such as acetate have been formed in the cathode chamber. There are several suggested pathways for bioelectrochemical CH_4_ production. Apart from CH_4_ production, *via* direct or indirect electron transfer, formation of other products could have occurred at the MEC cathode. For instance, H_2_ can be produced either electrochemically or bioelectrochemically and can be further used for the formation of CH_4_, acetate or formate. Moreover, CH_4_ can be also produced in a second step *via* bioelectrochemically produced acetate (Van Eerten-Jansen et al., 2015). However, during pure CO_2_ flushing no acetate formation was detected in the catholytes of MECs.

### Energetic evaluation

Table 4 summarizes the calculated energetic parameters for each experimental condition. The energy efficiency (ηE) was 48% and 49% for MEC1 at +400 mV and +300 mV vs. Ag/AgCl, respectively, and in both cases pure CO_2_ was used for methanation. These results are comparable with a previous study of a CH_4_ producing MEC for biogas upgrading at anodic potentiostatic control, where an energy efficiency of 52% was reported (Zeppilli et al., 2019). However, when exhaust gas was used for flushing MEC2 cathode, the energy efficiency dropped to 6% with SMO-1 and to 19% with SMO-2. This decrease may be related to the lower CH_4_ production rates of the biocathode due to the presence of inhibiting O_2_ concentrations in the exhaust gases. Furthermore, the energy consumptions for COD and CO_2_ removal were calculated. The energy consumptions for CO_2_ removal in MEC1 at +300 mV (20 kWh Nm^−^³) and +400 mV (20 kWh Nm^−^³) vs. Ag/AgCl were considerably higher compared to a previous reported one (4.27 kWh Nm^−^³) of a MEC for ammonium recovery and biogas upgrading at an applied anode potential of +200 mV vs. SHE (Zeppilli et al., 2021). If SMO-1 and SMO-2 were used for cathode flushing, the energy consumptions increased to 167 kWh Nm^−^³ and 52 kWh Nm^−^³, respectively, due to the inhibited transformation of CO_2_ into CH_4_. The energy input for CH_4_ formation with MEC1 (using pure CO_2_) was 20 kWh Nm^−^³, which is in line with a previous reported value of 19 kWh m^−^³ CH_4_ from Geppert et al. (Geppert et al., 2016). The higher energy consumption of the Sabatier process (26–35 kWh m^−^³ CH_4_), compared to the energy inputs obtained from the literature and confirmed by the performed experiments, profiles bioelectrochemical methanation as an attractive rival compared to Sabatier when pure CO_2_ is used (Geppert et al., 2016).

**TABLE 4:** Comparison of energetic parameters for all experimental conditions.

Energetic Parameters	MEC1		MEC2	
	+400 mV vs. Ag/AgCl	+300 mV vs. Ag/AgCl	SMO-1	SMO-2
E_Cell_ [V]	−1.53	−1.43	−1.62	−1.51
ηE [%]	48	49	6	19
kWh kg^−1^ COD	6.2	5.8	6.3	6.0
kWh Nm^−^³ CO_2_	20	20	167	52

The results led to the conclusion that methanation of SMO is in principle feasible in a MEC. However, due to inhibition of methanogens, as a consequence of the high O_2_ concentrations, CH_4_ production as well as energy efficiency decreased significantly, whereas the energy consumption for CO_2_ removal increased considerably. Possibilities to separate CO_2_ or O_2_ from exhaust gases are *e.g.*, adsorption or membrane separation (Dessì et al., 2021). In a next step the bioelectrochemically transformed CO_2_ from SMO could be used directly as a CH_4_ source in different industries, as for example the steel sector. For instance, this CH_4_ may be used as an alternative reducing agent in the blast furnace for the partial substitution of coke. This will cause a reduction of the CO_2_ emissions, due to the lower footprint of biological produced CH_4_ in comparison with coke (Remus et al., 2013). Furthermore, these observations may be interesting for other industry sectors, where exhaust gases with lower O_2_ concentrations are produced, which may allow its direct usage in the MEC without any previous separation step.

### Analysis of microbial community on electrode biofilms

**FIGURE 5:**
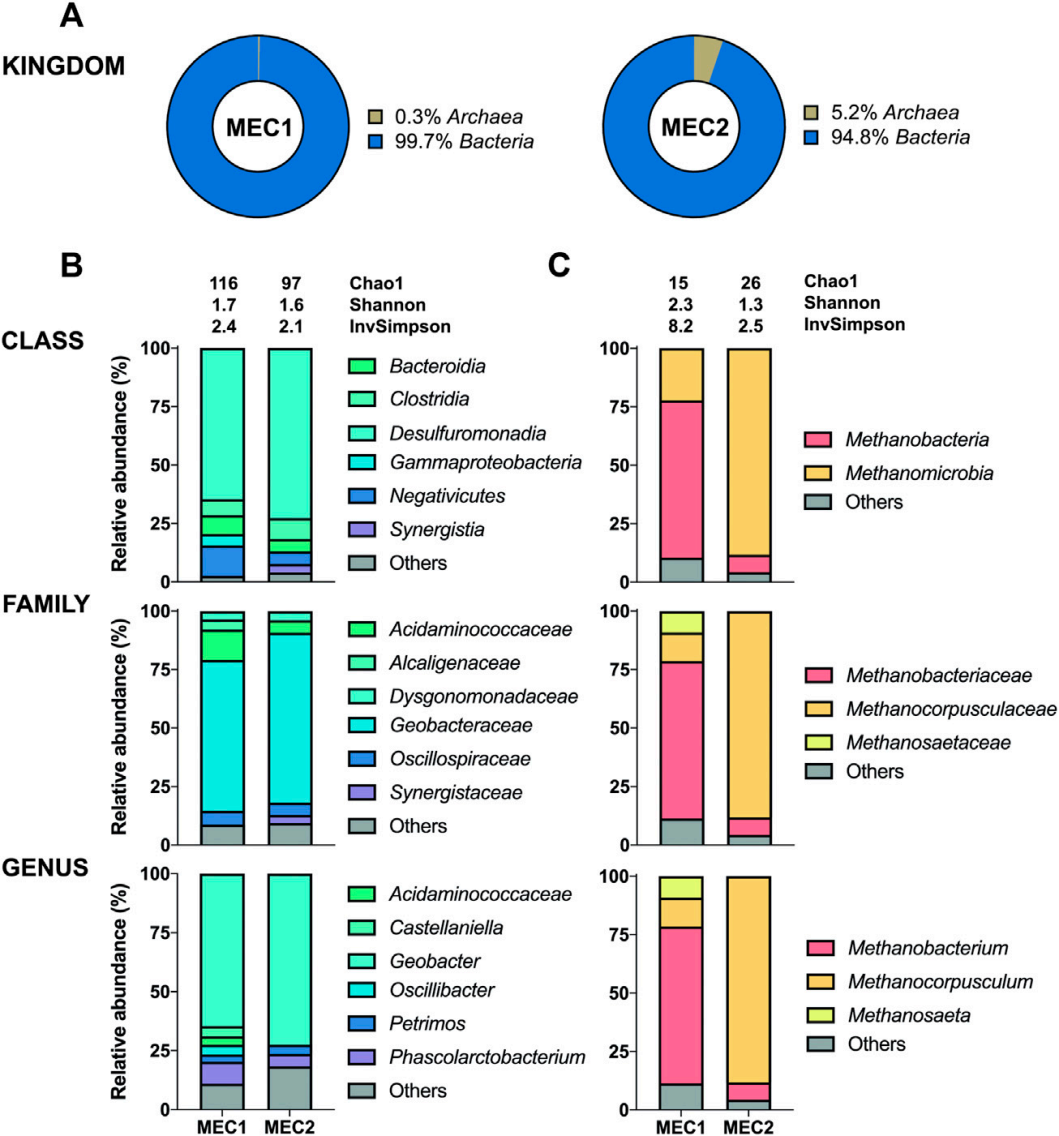
The enriched microbial communities of anodic biofilms in MECs. **(A)** Representation of prokaryotes determined by the 16S sequencing. Taxonomic profiles of bacteria **(B)** and methanogenic archaea **(C)** were set at the class, family, and genus ranks. Bacterial representation was determined by the 16S sequencing and methanogenic archaea by the *mcrA* sequencing. Only representatives with a relative abundance >3% in at least one condition are shown. Alpha diversity was estimated by the following indices: Chao1, Shannon, and Inverse Simpson. Detailed information is given in Supplementary Table S4.

**FIGURE 6:**
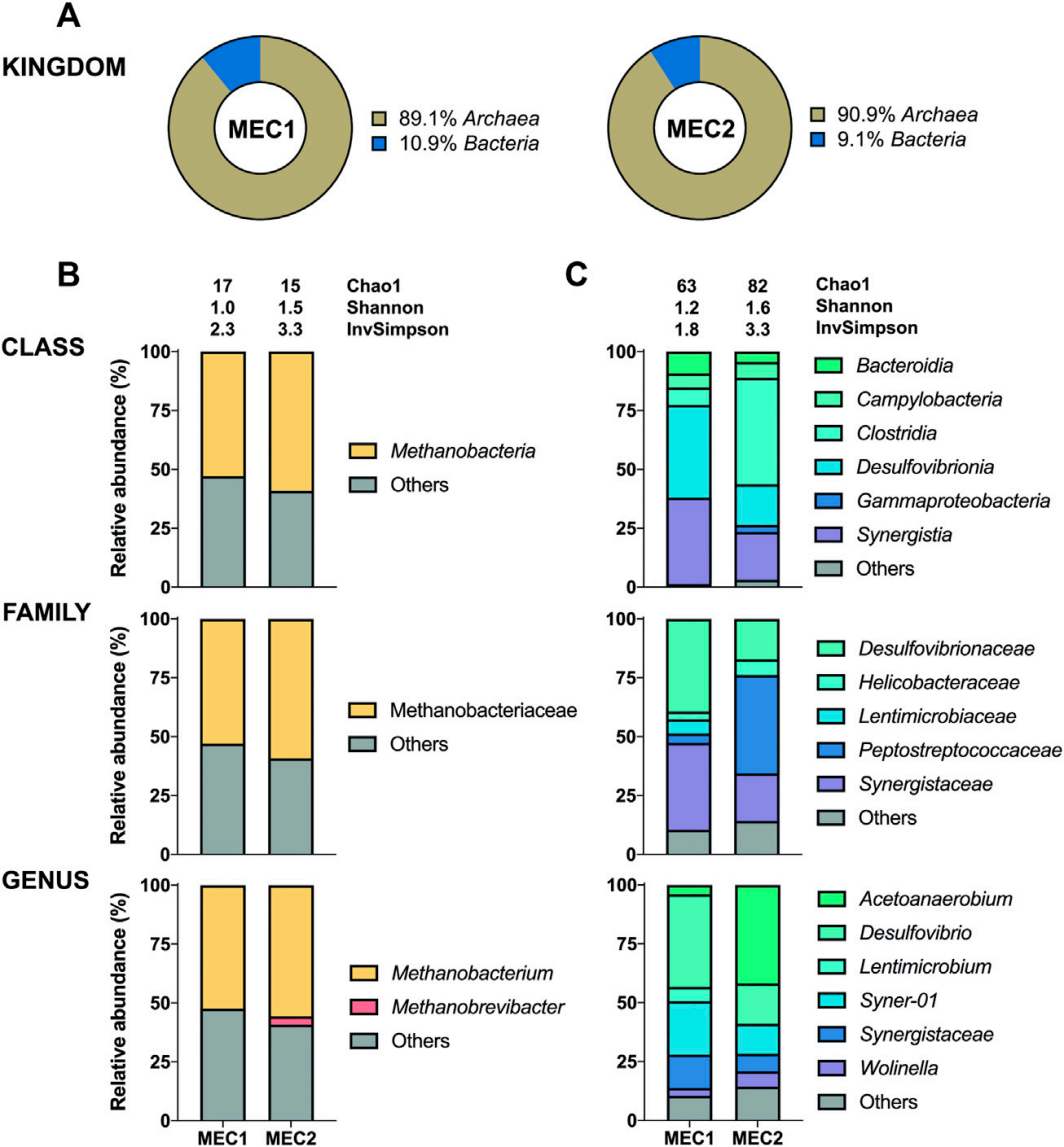
The enriched microbial communities of cathodic biofilm in MECs. **(A)** Representation of prokaryotes determined by the 16S sequencing. Taxonomic profiles of bacteria **(C)** and methanogenic archaea **(B)** were set at the class, family, and genus ranks. Bacterial representation was determined by the 16S sequencing and methanogenic archaea by the *mcrA* sequencing. Only representatives with a relative abundance >3% in at least one condition are shown. Alpha diversity was estimated by the following indices: Chao1, Shannon, and Inverse Simpson. Detailed information is given in Supplementary Table S4.

The 16S sequencing of the biofilms in MECs revealed a high proportion of bacteria adhering to anodes (95–100%) (Figure 5A), whereas archaea adhered predominantly to cathodes (89–91%) (Figure 6A). The distribution of bacteria was further divided into different taxonomic ranks based on the 16S sequencing (Figures 5B, 6C). Considering the significant proportion of archaea in CH_4_-producing cathodic biofilms, additional *mcrA* sequencing was performed to determine more accurately the distribution of methanogenic archaea into different taxonomic ranks (Figure 6B). For a comprehensive analysis, non-CH_4_-producing anodic biofilms were also analyzed using *mcrA* sequencing (Figure 5C).

*Geobacter* was the predominant genus adhered to MEC1 (64.6%) and MEC2 (72.5%) bioanodes, one of the prominent representatives using electrodes as electron acceptors for anaerobic respiration (Bond and Lovley, 2003). This genus can produce electrons and transfer them through conductive pili to the anode, whereas many other exoelectrogens need direct contact with the electrode (Logan et al., 2019). In particular, *Geobacter sulfurreducens* is known to produce very high current densities (Yi et al., 2009). The second most abundant genus attached to both bioanodes was *Phascolarctobacterium* with 9.3% (MEC1) and 5.3% (MEC2). This genus has also been identified in microbial fuel cells of other studies (Borole et al., 2009; Liu et al., 2013). Interestingly, *Oscillibacter* (4.2%), *Castellaniella* (4.3%), and *Acidaminococcaceae* (3.6%) were only identified at MEC1 bioanode, resulting in a higher biodiversity compared to MEC2. *Castellaniella* has been previously detected in the anodic biofilm and may contribute to electricity generation (Sun et al., 2011). In addition, differences in archaea abundance were observed between MEC1 and MEC2 bioanodes with 0.3% (MEC1) and 5.2% (MEC2).

The anodic biofilm in MEC1 was predominated in terms of archaea by *Methanobacterium* (67.2%), whereas *Methanocorpusculum* (88.2%) was predominant in anodic MEC2 biofilm. *Methanosaeta* (9.2%), present only in MEC1, can convert acetate, the primary carbon source in the anodes of both MECs, to CH_4_ but cannot use H_2_ or formate as an electron source for methanation (Smith and Ingram-Smith, 2007). Moreover, *Methanosaeta* spp*.* and *Geobacter* spp. can synergize through electron exchange, referred to as direct interspecies electron transfer (Rotaru et al., 2014; Zhao et al., 2015; Lovley, 2017). Differences in the anodic archaea population of MEC1 and MEC2 may be attributed to O_2_ diffusion across the membrane, since the cathode of MEC2 was flushed with SMO-1 containing of 4.6 vol% O_2_. O_2_ diffusion mechanisms from the anode to the biocathode were previously mentioned as a causer for parasitic reactions such as direct O_2_ reduction, leading to an increased current production but lower Coulombic efficiencies (Van Eerten-Jansen et al., 2012; Batlle-Vilanova et al., 2017). Therefore, O_2_, introduced by flushing the cathode chamber of MEC2 with SMO-1, may have diffused the opposite direction from the cathode to the anode, and has probably caused a shift in the archaea population of MEC2 if compared to MEC1.

*Methanobacterium* was found to be the dominant archaeon in CH_4_-producing cathodic biofilms (52.5% in MEC1 and 55.8% in MEC2). *Methanobacterium* is a typical member of biofilms attached to cathodes which can perform methanogenesis by direct electron uptake (Siegert et al., 2015). One of the prominent species performing electromethanogenesis is *Methanobacterium palustre* (Cheng et al., 2009). Genus *Methanobrevibacter* (3.4%) was only identified in the MEC2 biofilm. In a previous study this obligate anaerobic archaea mainly dominated cathodes with catalysts, such as platinum, supporting abiotic H_2_ production (Siegert et al., 2015). *Methanobrevibacter* is a strictly hydrogenotrophic archaeon that uses only H_2_ and CO_2_ to produce CH_4_, whereas *Methanobacterium* can also ferment acetate, ethanol and methanol (Cai et al., 2022). The use of SMO-1, to flush the biocathode of MEC2, probably affected the biofilm composition leading to a higher archaea diversity in MEC2. Furthermore, a high proportion of other uncharacterized species (41–48%) was observed in both biocathodes. Also, the presence of *Methanobrevibacter* in MEC2 may have been influenced by flushing with SMO-1 as the H_2_ concentration in the cathodic headspace have increased by three times during this experiment, which may have enhanced the growth of hydrogenotrophic *Methanobrevibacter*.

The most abundant bacteria were *Desulfovibrio* (39.3%) at MEC1 and *Acetoanaerobium* (41.8%) at MEC2 biocathodes. The anaerobic genus *Acetoanaerobium* produces acetate by metabolizing H_2_ and CO_2_ (Sleat et al., 1985), whereas *Desulfovibrio* spp. are well known for producing H_2_ when attached to an electrode surface (Jafary et al., 2015). In a previous study (Zheng et al., 2021) interspecies electron transfer between *Methanobacterium* and *Desulfovibrio* was studied, suggesting that *Methanobacterium* spp. can actively accept electrons from electron donating *Desulfovibrio* spp. . If strictly anaerobic *Methanobacterium* got inhibited due to cathodic SMO-1 flushing of MEC2, this may have influenced the syntrophic co-culture of *Methanobacterium* and *Desulfovibrio,* and possibly led to a shift in the bacterial community composition and suppressed genus *Desulfovibrio*, as observed when bacterial communities of MEC1 and MEC2 cathodes are compared, in which *Desulfovibrio* and *Acetoanaerobium* have predominated, respectively. In addition, the genera *Syner-01,* *Synergistaceae*, and *Wolinella* were present in MEC1 and MEC2 biocathodes, whereas the genus *Lentimicrobium* (6.1%) was found only in MEC1.

## Conclusion

This study investigated for the first time the CH_4_ production of a fully biocatalyzed MEC using SMO for flushing the biocathode. Two MECs, consisting of organic substrate-oxidizing bioanodes and CH_4_-producing biocathodes, were operated by applying a constant anode potential of +300 mV or +400 mV vs. Ag/AgCl. MEC1 served as a control during the experiments, whereas MEC2 was used for tests with SMO. Two exhaust gases with different compositions were examined. Higher O_2_ concentrations likely have reduced the rate of CH_4_ production during MEC2 operation due to the inhibition of anaerobic methanogens which have colonized the biocathode surface. However, it was noticeable that methanogenesis was still occurring in the presence of O_2_ from SMO-1 even though at lower CH_4_ production rates than before. Upon subsequent methanation by application of pure CO_2_, the biofilm successfully recovered from inhibition and achieved a CH_4_ production rate of 10.8 L m^−2^ d^−1^. Therefore, separating O_2_ from steel mill off-gas seems to be essential to increase CO_2_ recycling and CH_4_ production rates, as well as to decrease the energy consumption for CO_2_ removal in CH_4_ producing MECs. Furthermore, the effect on the CH_4_ production rate, by reducing the anode potential from +400 to +300 mV vs. Ag/AgCl, was investigated in MEC1. The CH_4_ production of MEC1 remained constant despite the reduced anode potential but COD removal efficiency and current density increased. The microbial diversity of bacteria and archaea, attached to both MEC bioelectrodes, was also investigated and compared with each other. The genus *Geobacter* predominated the anodic biofilms, whereas the genus *Methanobacterium* was the most abundant one in cathodic biofilms. Differences in microbial community compositions of MEC1 and MEC2 bioelectrodes, which may be related to SMO flushing, have been identified and discussed.

## Data Availability

The datasets presented in this study can be found in online repositories. The names of the repository/repositories and accession number(s) can be found in the article/Supplementary Material.
